# Supplementary material for: A developmental biliary lineage program cooperates with Wnt activation to promote cell proliferation in hepatoblastoma
Source: Nat Commun. 2024 Nov 20;15:10007. doi: 10.1038/s41467-024-53802-4 (PMC11579301; doi:10.1038/s41467-024-53802-4)
Supplement: Supplementary file 1 — Supplementary Information [file 41467_2024_53802_MOESM1_ESM.pdf]

SUPPLEMENTARY INFORMATION

A developmental biliary lineage program cooperates with Wnt activation to promote cell proliferation in hepatoblastoma

Peng V. Wu[1,2,3,4,5,6]*, Matt Fish[1,2,3], Florette K. Hazard[7,8], Chunfang Zhu[7], Sujay Vennam[7], Hannah Walton[1,2,3,9], Dhananjay Wagh[10], John Coller[10], Joanna Przybyl[7,11,12], Maurizio Morri[13,14], Norma Neff[14], Robert B. West[7], and Roel Nusse[1,2,3]*

[1] Howard Hughes Medical Institute, Stanford University School of Medicine, Stanford, CA 94305, USA
[2] Department of Developmental Biology, Stanford University School of Medicine, Stanford, CA 94305, USA
[3] Institute for Stem Cell Biology and Regenerative Medicine, Stanford University School of Medicine, Stanford, CA 94305, USA
[4] Department of Pediatrics, Stanford University School of Medicine, Stanford, CA 94305, USA
[5] Current affiliation: Division of Oncology, Cincinnati Children's Hospital Medical Center, Cincinnati, OH 45229, USA
[6] Current affiliation: Department of Pediatrics, University of Cincinnati College of Medicine, Cincinnati, OH 45229, USA
[7] Department of Pathology, Stanford University School of Medicine, Stanford, CA 94305, USA
[8] Current affiliation: Department of Pathology and Laboratory Medicine, University of California Davis School of Medicine, Sacramento, CA 95817 USA
[9] Current affiliation: Department of Population Health, NYC Health + Hospitals, New York, NY 10004 USA
[10] Stanford Genomics, Stanford University, Stanford, CA 94305, USA
[11] Current affiliation: Department of Surgery, McGill University, Montreal, H4A 3J1 QC, Canada
[12] Current affiliation: Cancer Research Program, The Research Institute of the McGill University Health Centre, Montreal, H4A 3J1 QC, Canada
[13] Chan Zuckerberg Biohub, Stanford, CA 94305, USA
[14] Current affiliation: Altos Labs, Redwood City, CA 94065, USA

*Correspondence to: Peng.Wu@cchmc.org and rnusse@stanford.edu

Supplementary Table S1 (related to Fig. 1).
Characteristics of hepatoblastoma specimens used for Smart-3SEQ

Pt	Sex	Histology	Prior Chemo	FGF19	
				Smart-3SEQ	In situ
**LCM-1**	F	Fetal & Embryonal	N	-	n.d
**LCM-2**	M	Predominant Embryonal	N	+	n.d.
**LCM-3**	F	Mixed	Y	+	+
**LCM-4**	M	Mixed	Y	-	n.d
**LCM-5**	F	Mixed	Y	+	-
**LCM-6**	M	Fetal & Embryonal	N	+	+
**LCM-7**	M	Mixed	N	+	+
**LCM-8**	M	Fetal & Embryonal	N	+	+
**LCM-9**	F	Mixed	Y	-	n.d
**LCM-10**	F	Fetal & Embryonal	N	+	+
**LCM-11**	F	Fetal & Embryonal	N	+	+
**LCM-12**	F	Fetal & Embryonal	N	+	n.d
**LCM-13**	M	Fetal & Embryonal	N	-	n.d
**LCM-14**	M	Fetal & Embryonal	N	-	n.d
**LCM-15**	F	Mixed	Y	-	n.d
**LCM-16**	F	Mixed	Y	-	n.d
**LCM-17**	M	Mixed	Y	-	n.d

Characteristics of the patients and tumors reported are from the date of the specimen. n.d. = not determined.

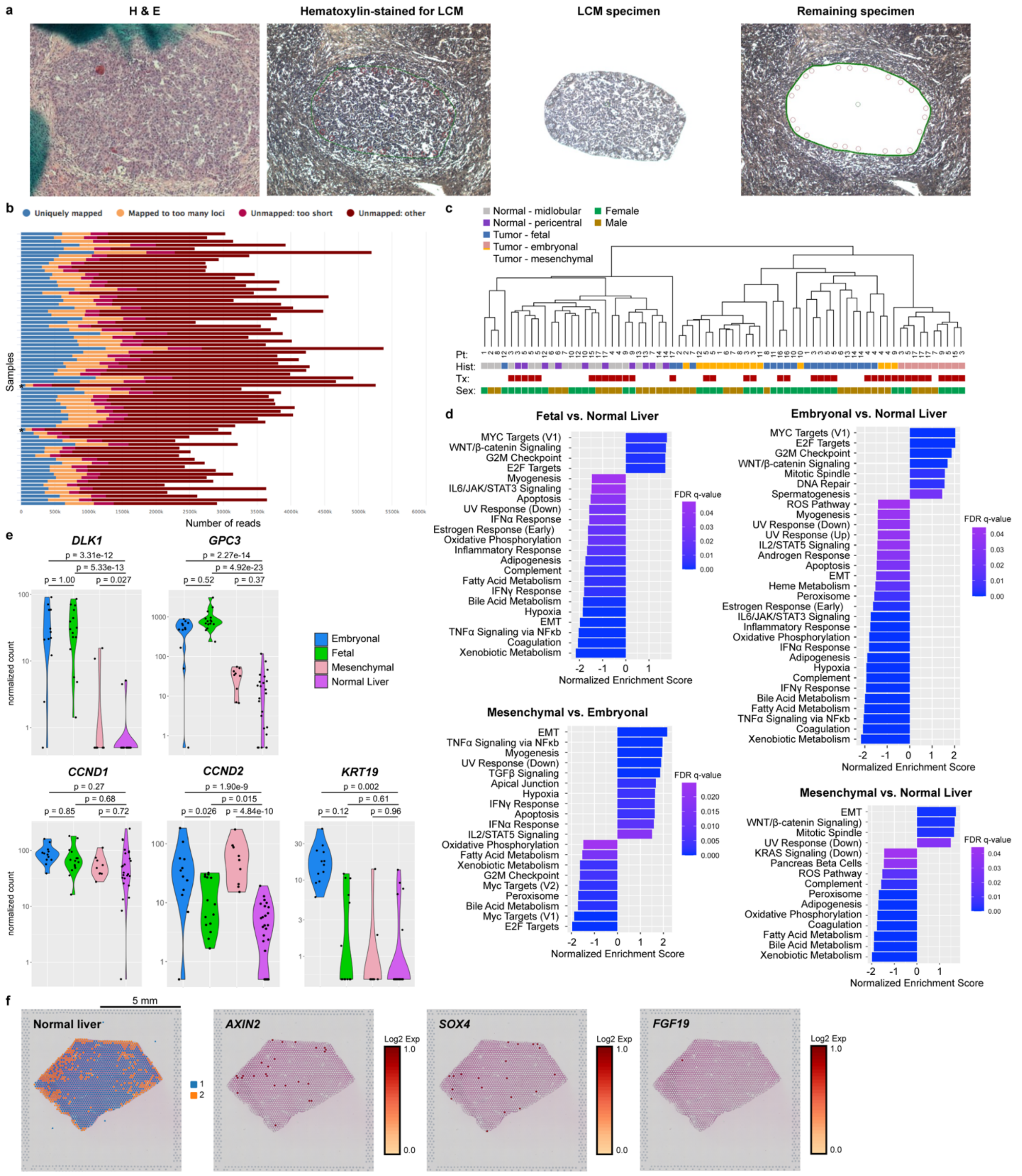

Supplementary Fig. S1 (related to Fig. 1). a) Example of specimen used for laser capture microdissection, from left: stained with hematoxylin and eosin (H&E), stained with hematoxylin for dissection, after laser capture microdissection, and remaining specimen following microdissection. b) Read alignment of samples assayed by Smart-3SEQ, using

STAR. * indicates samples with poor quality that were excluded from further analysis. c) Unsupervised hierarchical clustering of results from Smart-3SEQ. d) Top hallmark pathways differentially expressed between Fetal vs. Normal Liver, Embryonal vs. Normal liver, Mesenchymal vs. Normal Liver and Mesenchymal vs. Embryonal components of hepatoblastoma (FDR < 0.05), using gene set enrichment analysis (GSEA) on pre-ranked gene lists obtained from DESeq2. e) Normalized counts of *DLK1*, *GPC3*, cyclins *CCND1* and *CCND2*, and cholangiocytic marker *KRT19*. Adjusted p-values determined by two-tailed Wald test with Benjamini-Hochberg adjustment for multiple hypothesis testing. Blue: embryonal, green: fetal, pink: mesenchymal, purple: normal liver. f) Clusters identified by 10x Visium spatial transcriptomics on normal liver and spatial expression of *AXIN2*, *SOX4*, and *FGF19*. Source data are provided as a Source Data file.

Supplementary Table S2 (related to Fig. 1). Signature Genes for Different Hepatoblastoma Histologies based on Smart-3SEQ

Fetal	Embryonal	Mesenchymal	Fetal-Specific	Embryonal-Specific	Mesenchymal-Specific
PEG10	NKD1	GNAS	AZGP1	AMBN	SPARC
GPC3	GNAS	SPARC	G6PC	MDFI	MEF2C
BCAM	BCAM	MMP14	C1S	MEX3A	BGN
NKD1	PEG10	COL11A2	CYP4F3	VCAN	COL3A1
PEG3	ROBO1	ROBO1	BAAT	MMP16	TIMP2
FRRS1L	RP11-51O6.1	FKBP10	HP	DACH1	COL5A1
DUSP9	DKK1	MAP1B	ADH1B	SOX4	PDGFRA
MEG3	PEG3	MSX1	ABCA6	TYRO3	COL1A1
ACSL4	RPS7P1	CKAP4	PCK1	FBLN2	DCN
ROBO1	MAP4K4	APCDD1	SLC51A	LNPEP	DNM3OS
DLK1	HMGA2	UCHL1	HSD17B6	IBSP	CD9
IGF2BP1	APCDD1	S100A4	C1R	NAP1L1	COL1A2
ARID3A	MEX3A	MEF2C	ITIH3	RCN2	ANXA1
MAGED1	IGDCC3	UNC5B	CYP3A4	TPBG	MGP
TRIM71	FKBP10	ZFHX3	CYP8B1	GNAS	IGFBP7
ITGA6	ARID3A	SERPINH1	F9	CXCL14	IFI6
AFP	TRIM71	MDFI	SERPING1	NPTX1	PLXDC2
IGDCC3	NAP1L1	SP6	CPT1A	LAPTM4B	ELN
SQLE	IGF2BP1	COLEC12	HRG	CRABP2	ITM2B
GNG4	UGGT1	COL15A1	CYP27A1	FKBP10	CALM1
TMEM246	MDK	COL1A2	C6	TEAD2	PLS3
EPCAM	FRRS1L	PLS3	CYP2C9	PTCH1	NFIC
PTP4A3	HELLS	S100A1	HPR	POSTN	CYBRD1
PLPPR1	GPC3	PLXNA1	AOX1	MARCKSL1	TIMP3
SNHG23	RP11-864N7.2	COL9A3	C8G	SOHLH2	ANO1
RP11-51O6.1	AXIN2	MAGED2	ADH1C	MSX1	FXYD6
GNAS	TCF3	MMP11	CYP4A22	FREM2	AHNAK
COL2A1	MSX1	PLD3	LEAP2	HNRNPA2B1	CD81
SNAP25-AS1	PLPPR1	OXCT1	SULT2A1	ILF3	ENG
DKK1	RPS3	COL1A1	UGT2B10	TUBBP1	COL6A1
PLVAP	LAPTM4B	NOTCH3	CYP2E1	RP11-278C7.1	EGR1
CACNB4	DPEP1	ANXA5	CES2	CKAP4	ZCCHC24
APCDD1	EPCAM	ANGPTL2	ORM2	CPXM1	JUNB
MAP4K4	TRIM24	PPT1	HMGCS2	MYCN	VIM
KCNU1	DUSP9	ATP8B2	APOA5	PHF6	COL12A1
FAM3B	MAGED1	KIDINS220	TAT	XRCC5	ANXA5
UGGT1	NT5DC2	CPE	APCS	HNRNPU	PPP1R12B
TRIM24	LEF1	GSN	APOC3	SLC34A2	IDS
SLC22A11	RPL13P2	UACA	CCL16	DBN1	LPAR1
NPNT	RP11-771F20.1	NES	IGFBP1	GPR161	AEBP1
ONECUT2	RP5-1056L3.3	COL5A2	CYB5A	BIRC5	TAGLN
MBNL3	LRRC75A-AS1	ITM2B	ANGPTL3	TP53BP1	EMILIN1
EBF1	DLK1	EBF1	APOC1	WLS	KALRN
SP5	HDAC2	VIM	FMO3	FAM84A	C1R
HMGA2	MDFI	TIMP2	SLC22A7	AXIN2	MMP14
IKBKAP	PGC	DKK1	ACSM2A	ELMO1	ECM1
PABPC1	LIN28B	CUX1	AKR1C2	RASL11B	LMNA
LEF1	AFP	S100A6	PIPOX	VANGL2	FSTL1
COL15A1	MYCN	MAGED1	PCK2	HDAC2	COL8A1
MDK	COL2A1	LEF1	A2M	HMGA2	S100A6

Top 50 differentially expressed genes obtained by Smart-3SEQ and DESeq2 comparing fetal vs normal (Fetal), embryonal vs. normal (Embryonal), mesenchymal vs. normal (Mesenchymal), fetal vs. embryonal (Fetal-Specific), embryonal vs. fetal (Embryonal-Specific), mesenchymal vs. embryonal (Mesenchymal-Specific).

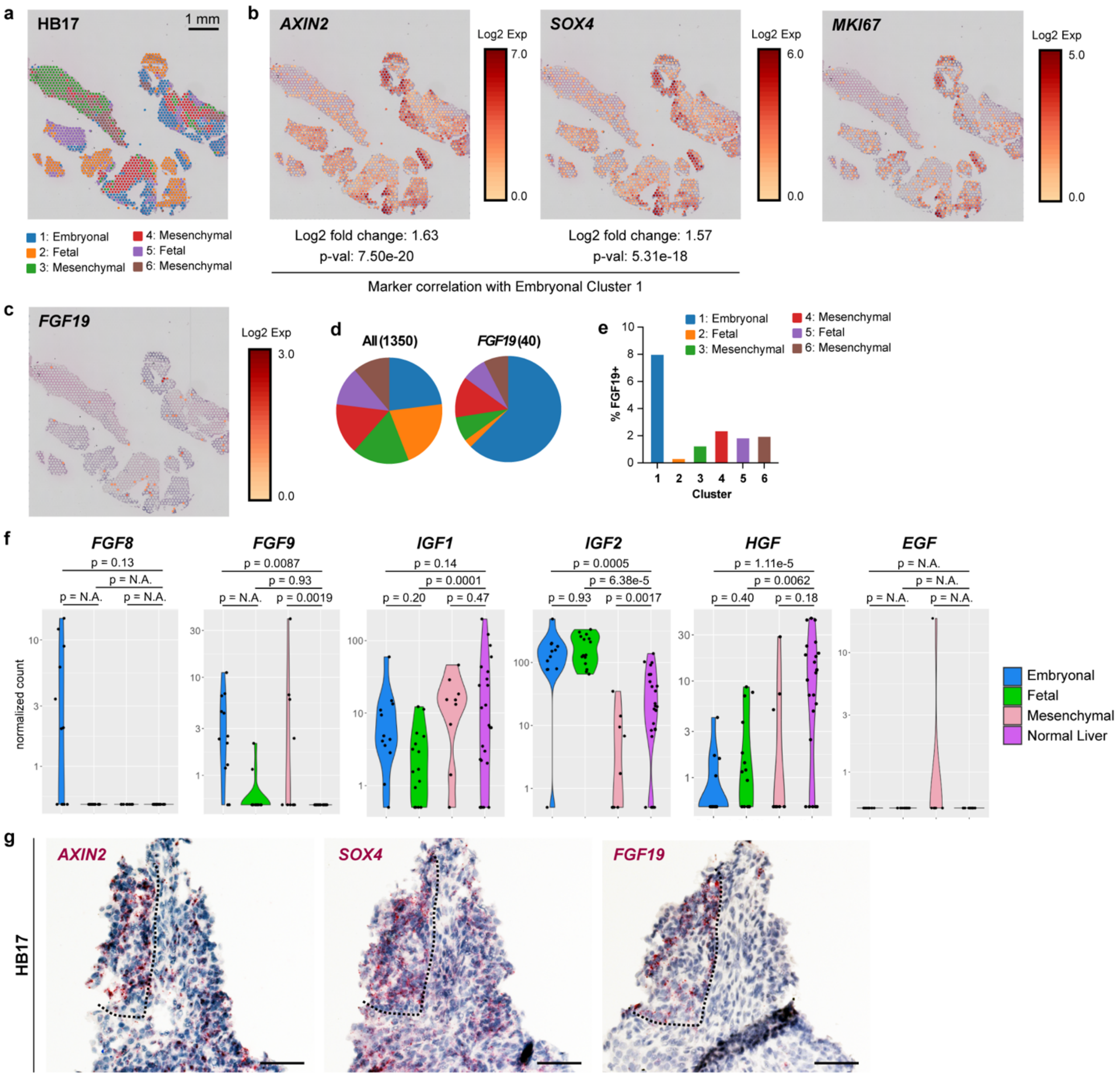

Supplementary Fig. S2 (related to Fig. 2). a) Clusters identified by 10x Visium spatial transcriptomics on primary hepatoblastoma HB17, assigned to fetal, embryonal, or mesenchymal histologies based on genes identified by Smart-3SEQ. b) Spatial expression of *AXIN2*, *SOX4*, and *MKI67* in HB17. Adjusted p-values determined in CellRanger by negative binomial test with Benjamini-Hochberg adjustment for multiple hypothesis testing. c) Spatial expression of *FGF19* in HB17. d) Pie charts showing distribution of all spots and all FGF19^{+} spots according to cluster. e) Quantification of FGF19^{+} spots/total spots in each cluster. f) Normalized counts of *FGF8*, *FGF9*, *IGF1*, *IGF2*, *MET*, and *EGF* detected by Smart-3SEQ. Adjusted p-values determined by two-

tailed Wald test with Benjamini-Hochberg adjustment for multiple hypothesis testing. Blue: embryonal, green: fetal, pink: mesenchymal, purple: normal liver. g) RNA in situ hybridization in serial sections of primary hepatoblastoma HB17 detecting *AXIN2*, *SOX4*, and *FGF19* in red. Black dashed lines outline cells expressing *FGF19*. Scale bar: 50 μm. Source data are provided as a Source Data file.

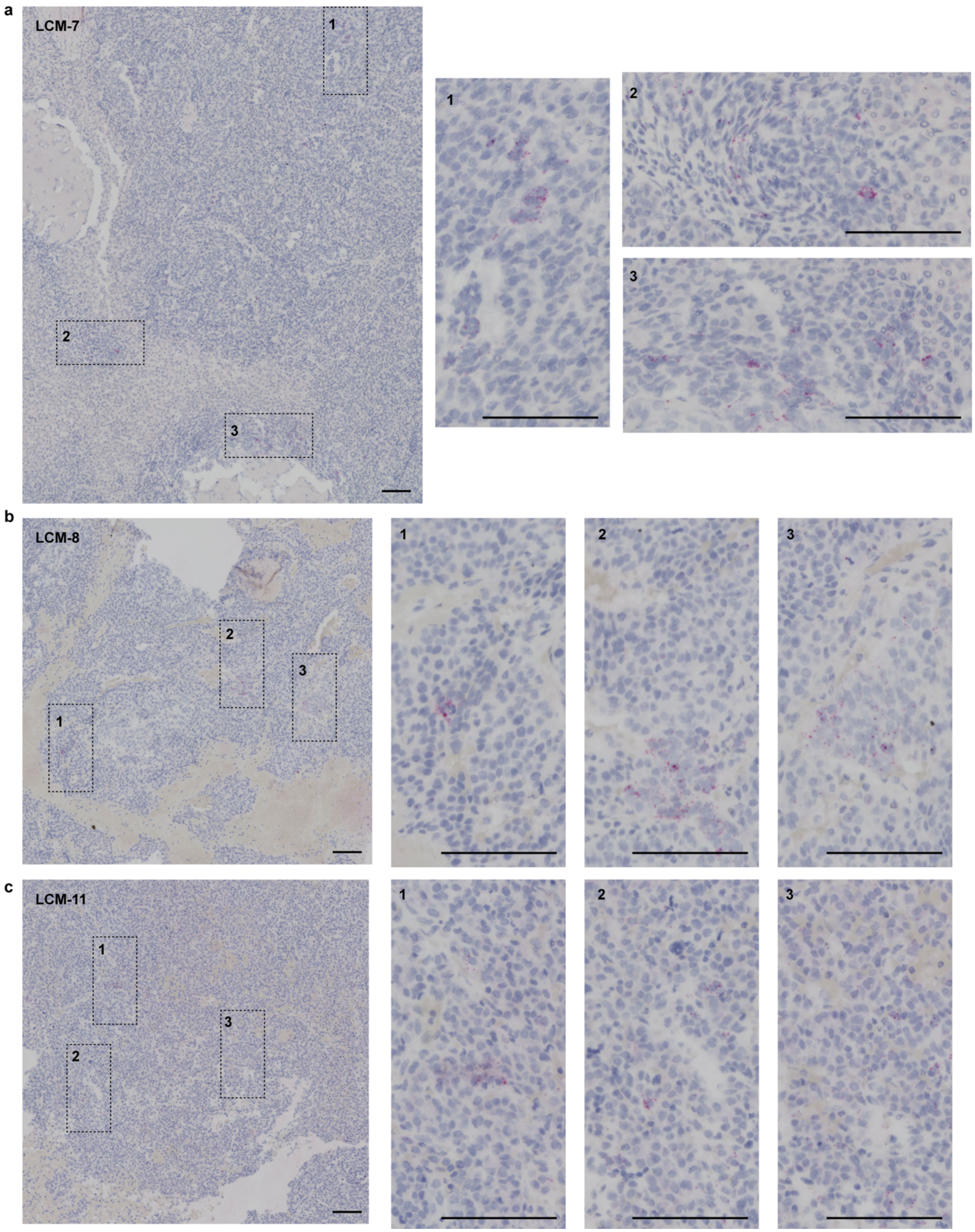

Supplementary Fig. S3 (related to Fig. 2). FGF19 expression in primary hepatoblastomas. RNA in situ hybridization detecting FGF19 (red) in primary

hepatoblastoma specimens used for Smart-3SEQ from the different patients as indicated: a) LCM-7, b) LCM-8, c) LCM-11. Black dotted rectangles show numbered regions that are magnified. Scale bar = 50 μm.

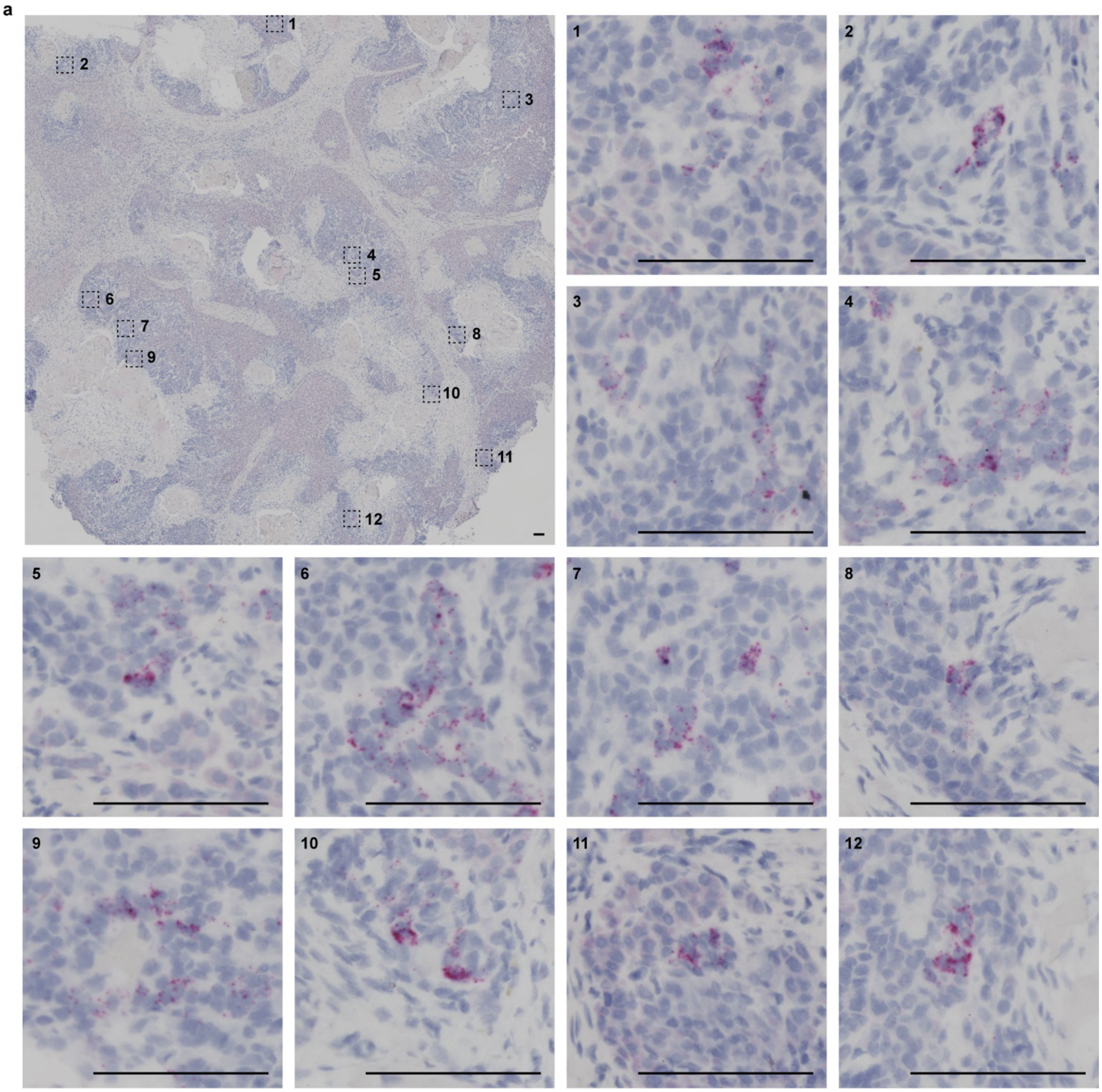

Supplementary Fig. S4 (related to Fig. 2). FGF19 expression in primary hepatoblastomas. a) RNA in situ hybridization detecting FGF19 (red) in the primary hepatoblastoma from which tumoroid HB4 was derived. Black dotted boxes show numbered regions that are magnified. Scale bar = 50 μm. Note: this is the same specimen used for spatial transcriptomics in Fig. 2b.

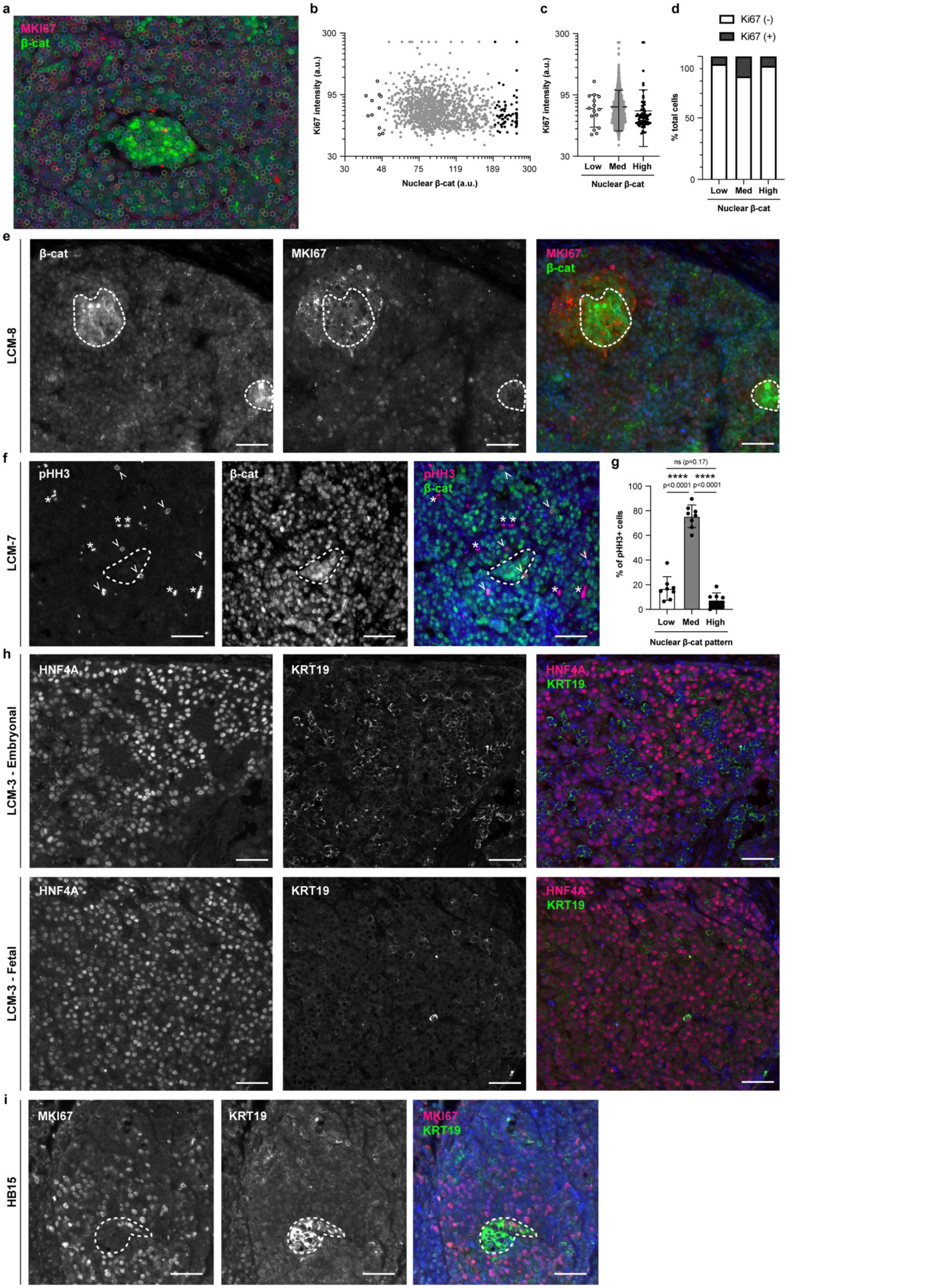
a
MKI67
β-cat
b
Ki67 intensity (a.u.)
Nuclear β-cat (a.u.)
c
Low
Med
High
Nuclear β-cat
d
Ki67 (-)
Ki67 (+)
% total cells
e
LCM-8
β-cat
MKI67
f
LCM-7
pHH3
g
ns (p=0.17)
p<0.0001
% of pHH3+ cells
Nuclear β-cat pattern
h
LCM-3 - Embryonal
HNF4A
KRT19
LCM-3 - Fetal
i
HB15

Supplementary Fig. S5 (related to Fig. 3). a) Representative image of co-immunofluorescence of MKI67 (magenta) and β-catenin (green) in HB15 with nuclear masks automatically drawn by TrackMate (ImageJ/Fiji plugin). b) Plot of Ki67 intensity within the nuclear masks in a) as a function of nuclear β-catenin intensity, colored by category: low (open circles), med (gray circles), and high (black circles). c) Distribution of Ki67 intensity comparing cells with low (open circles), med (gray circles), and high (black circles) nuclear β-catenin intensity from image in a). d) Percentage of $Ki67^{+}$ (intensity > 95 a.u) vs. $Ki67^{-}$ cells (intensity < 95 a.u.) in cells with low (open circles), med (gray circles), and high (black circles) nuclear β-catenin intensity from image in a). e) Co-immunofluorescence of MKI67 (magenta) and β-catenin (green), merged with DAPI (blue), in primary hepatoblastoma section from patient LCM-8. f) Co-immunofluorescence of phospho-histone H3 (pHH3) (magenta) and β-catenin (green), merged with DAPI (blue). White arrowheads indicate pHH3+ cells. White asterisks indicate non-specific staining of blood cells. i) Quantification of percentage of pHH3+ cells with different patterns of β-catenin staining as indicated. Mean and s.d., n=8 patient specimens, >60 pHH3+ cells scored for each experiment, ordinary one-way ANOVA, paired, with Tukey's correction for multiple comparisons. g) Co-immunofluorescence of HNF4A (magenta) and KRT19 (green), merged with DAPI (blue) in primary hepatoblastoma section from patient LCM-3, with representative images taken from regions of embryonal (top panels) and fetal (bottom panels) histology. h) Co-immunofluorescence of MKI67 (magenta) and KRT19 (green), merged with DAPI (blue). For all panels – scale bar: 50 μm, ****: p <0.0001, ns: not significant. Source data are provided as a Source Data file.

Supplementary Table S3 (related to Fig. 4). Characteristics of hepatoblastoma tumoroids

	Sex	Histology	Stage/Risk	Prior Chemotherapy	Specimen source	*CTNNB1* mutation	Other mutations (VAF)#	Status
**HB1**	M	Fetal & Embryonal	Relapsed/metastatic	Y	Lung metastasis	A21_G38del	n.d.	Deceased
**HB4**	M	Mixed (with teratoid)	Intermediate Risk (later relapsed)	Y	Resection	A5_S37del	*FGF4* P175S (48%)<br>*SMAD4* I525V (50%)	CR2
**HB6**	M	Mixed (with teratoid)	Relapsed	Y	Resection	D32N	*AR* A646 (99%)<br>*FGFR2* c1287+2A>G (38%)	Deceased
**HB7***	unknown	Fetal & Embryonal	Unknown	Y	Resection	S23_S33delinsP	n.d.	unknown
**HB12**	F	Fetal & Embryonal	Low Risk	N	Biopsy	G34V	*ARID1A* W1844ter (germline)<br>otherwise n.d.	CR1
**HB13**	M	Mixed (with teratoid)	Intermediate Risk	Y	Resection	G34R	*APC* I2341X (germline)<br>*FGF4* A29V (48%)	CR1
**HB14**	F	Mixed (with teratoid)	Intermediate Risk	N	Biopsy	A5_A80del	*GATA3* A396T (47%)	CR1
**HB15**	M	Mixed (with teratoid)	Low Risk (later relapsed)	Y	Resection	A5_A80del	*TSC2* V1034I (52%)<br>*APC* N2109H (49%)<br>*ATM* H231R (47%)	CR2
**HB16**	F	Fetal & Embryonal	High Risk (metastatic)	N	Biopsy	A5_A80del	*LRP1B* E71* (58%)<br>*BCR* V1107I (18%)<br>*CDKN1B* L120M (43%)<br>*SMARCA4* P913L (39%)<br>*BCR* T1018A (14%)<br>*NFE2L2* E79_E82del (25%)	Ongoing treatment
**HB17**	F	Mixed	Intermediate Risk	N	Biopsy	A5_A80del	*ARID1A* N106fs (13%)<br>*BTK* R492H (50%)<br>*ERBB2* P625L (46%)<br>*KDR* A772V (45%)<br>*FOXR2* S92N (41%)<br>*FGF4* A24T (35%)	CR1

Characteristics of the patients and tumors reported are from the date of the specimen. n.d. = not determined.
#Mutations identified from the Stanford Actionable Mutation Panel (STAMP) for Solid Tumors, an in-house NGS assay that covers 130 genes.
*Gift from Bruce Wang (UCSF), corresponds to patient 7 from Song et al. 2022

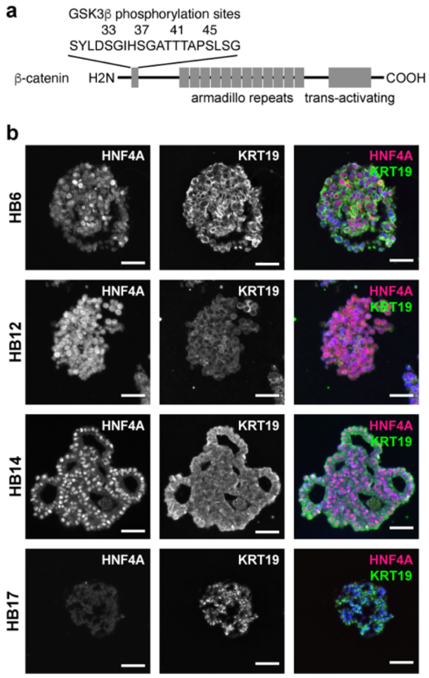

Supplementary Fig. S6 (related to Fig. 4). a) Domain structure of β-catenin showing the GSK3β phosphorylation sites within the region of exon 3 affected by mutations in the hepatoblastoma tumoroids. b) Immunofluorescence detecting HNF4A (magenta), KRT19 (green), and merged with DAPI (blue) in tumoroids from HB6, HB12, HB14, HB17 (late passage). Scale bar: 50 μm.

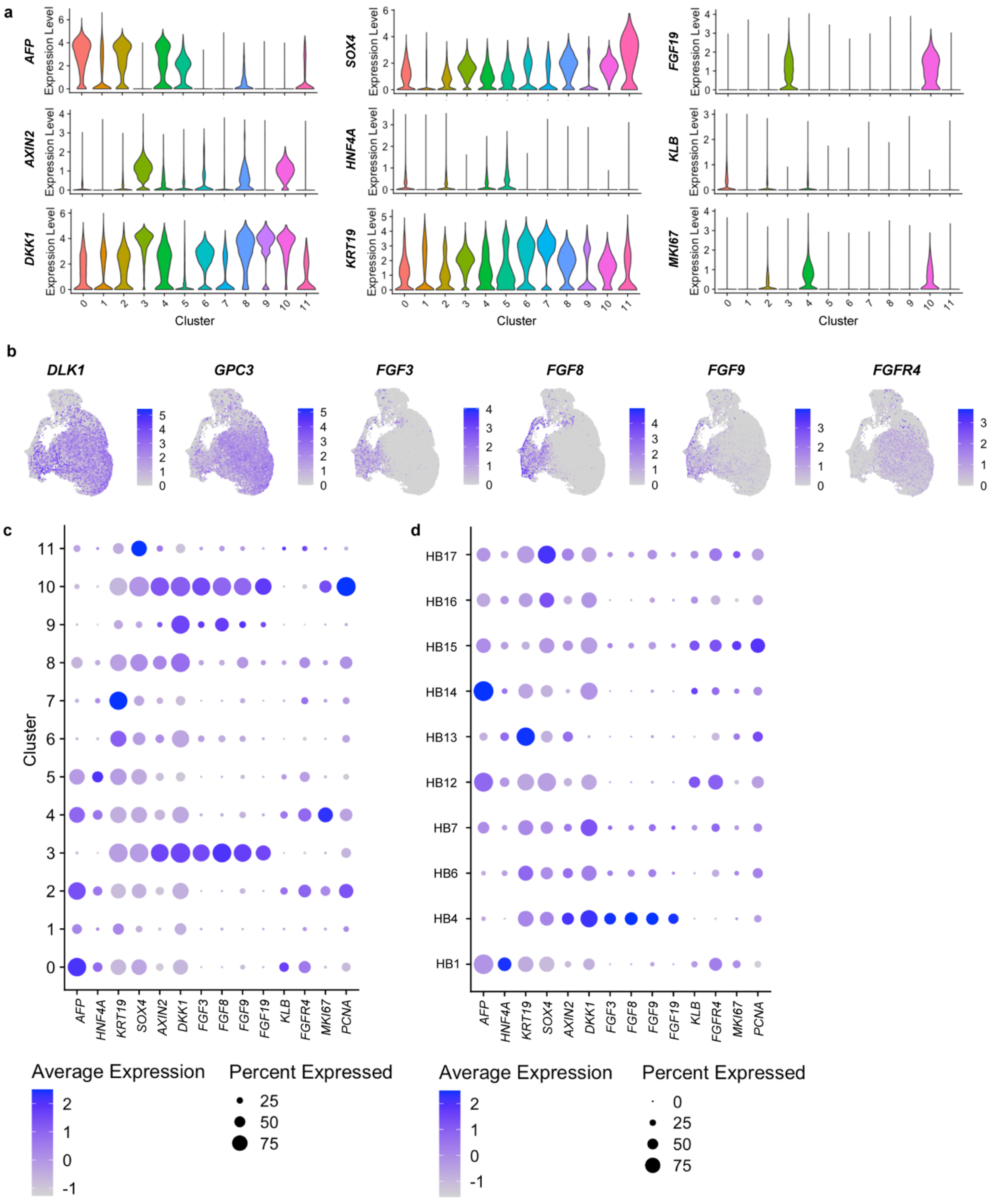

Supplementary Fig. S7 (related to Fig. 5). a) Violin plots showing relative gene expression of different hepatoblastoma genes across aggregated clusters from all tumoroids. b) UMAP plots showing relative gene expression for hepatoblastoma markers,

FGFs, FGFR4. Color scales indicate normalized expression obtained by the LogNormalize function in Seurat. c) Dot plot showing average gene expression and % of cells expressing various markers in different clusters. d) Dot plot showing average gene expression and % of cells expressing various markers in different HB tumoroids.

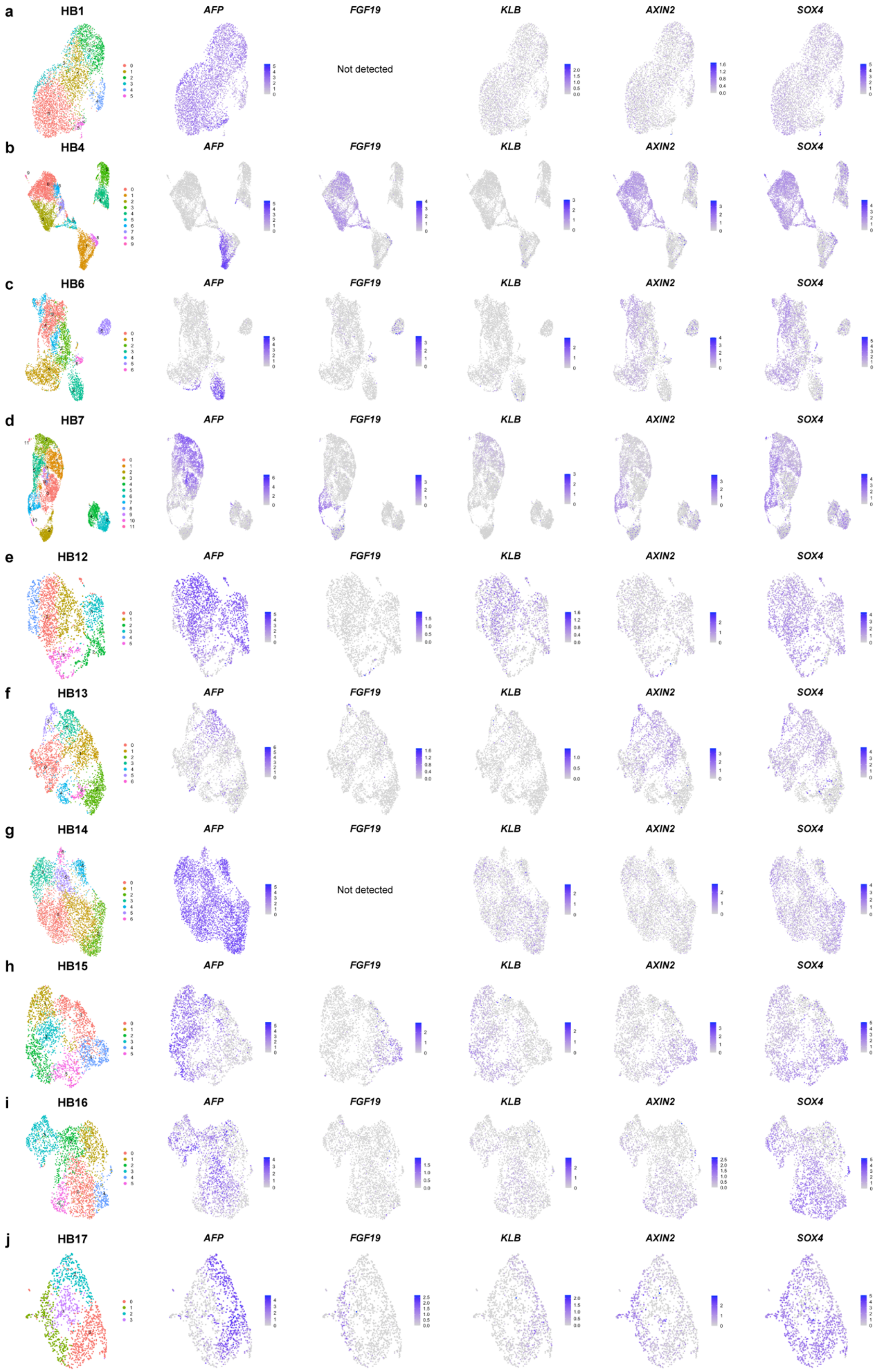
a HB1 AFP FGF19 KLB AXIN2 SOX4
Not detected
b HB4 AFP FGF19 KLB AXIN2 SOX4
c HB6 AFP FGF19 KLB AXIN2 SOX4
d HB7 AFP FGF19 KLB AXIN2 SOX4
e HB12 AFP FGF19 KLB AXIN2 SOX4
f HB13 AFP FGF19 KLB AXIN2 SOX4
g HB14 AFP FGF19 KLB AXIN2 SOX4
Not detected
h HB15 AFP FGF19 KLB AXIN2 SOX4
i HB16 AFP FGF19 KLB AXIN2 SOX4
j HB17 AFP FGF19 KLB AXIN2 SOX4

Supplementary Fig. S8 (related to Fig. 5). UMAP representation of scRNA sequencing results of hepatoblastoma tumoroids from 10 different patients analyzed separately, colored by cluster, and showing gene expression of *AFP*, *FGF19*, *KLB*, *AXIN2*, and *SOX4*. a) HB1, b) HB4, c) HB6, d) HB7, e) HB12, f) HB13, g) HB14, h) HB15, i) HB16, j) HB17. Color scales indicate normalized expression obtained by the LogNormalize function in Seurat.

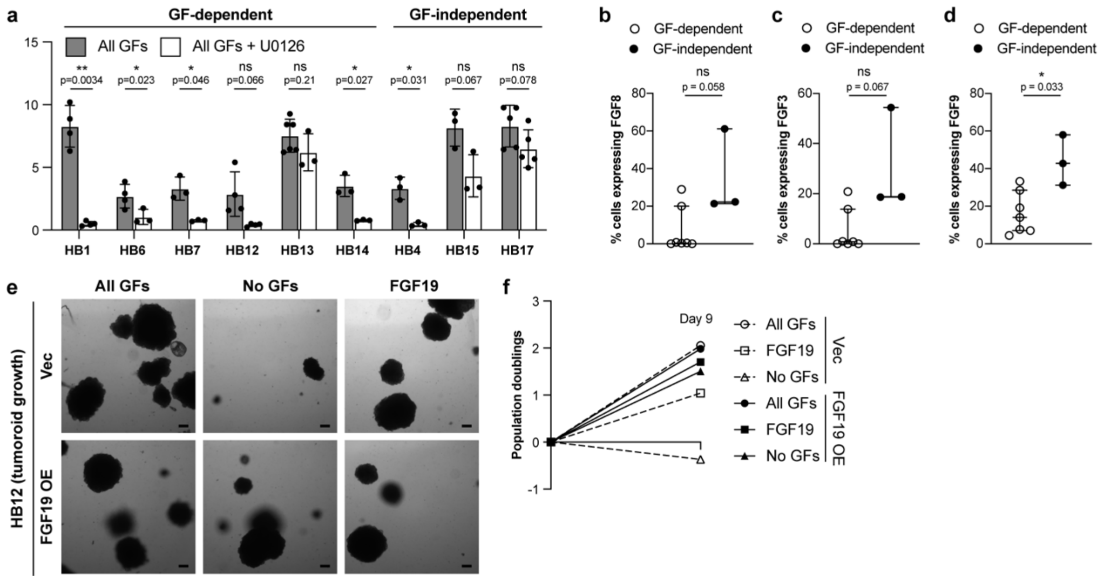

Supplementary Fig. S9 (related to Fig. 6). a) Quantification of colony formation assay of single cells from tumoroids growing for 2 weeks in media containing all GFs (EGF + FGF10 + HGF) or all GFs + MEK inhibitor U0126 (5 μM). Mean and s.d. for HB1 (n=4), HB6 (n=4), HB7 (n=3), HB12 (n=4), HB13 (n=6), HB4 (n=3), HB15 (n=3), HB17 (n=5), paired two-tailed student t-test except for HB6 and HB13 (unpaired two-tailed student t-test). Percentage of cells expressing b) FGF8, c) FGF3, and d) FGF9 determined by scRNAseq. Median and interquartile range, comparing GF-dependent (open circles, n=6) and GF-independent (filled circles, n=3) tumoroids by Mann-Whitney test. e) HB12 tumoroids transduced with lentiviral GFP vector or FGF19, at the end of 9 days after initially plating as intact tumoroids to a density of 50,000 cells/well and grown in all GFs (left), no GFs (middle), or FGF19 (right). Scale bar: 100 μm. f) Population doublings determined from n=1 experiment in e). For all panels – *: p<0.05, **: p<0.01, ns: not significant. Source data are provided as a Source Data file.

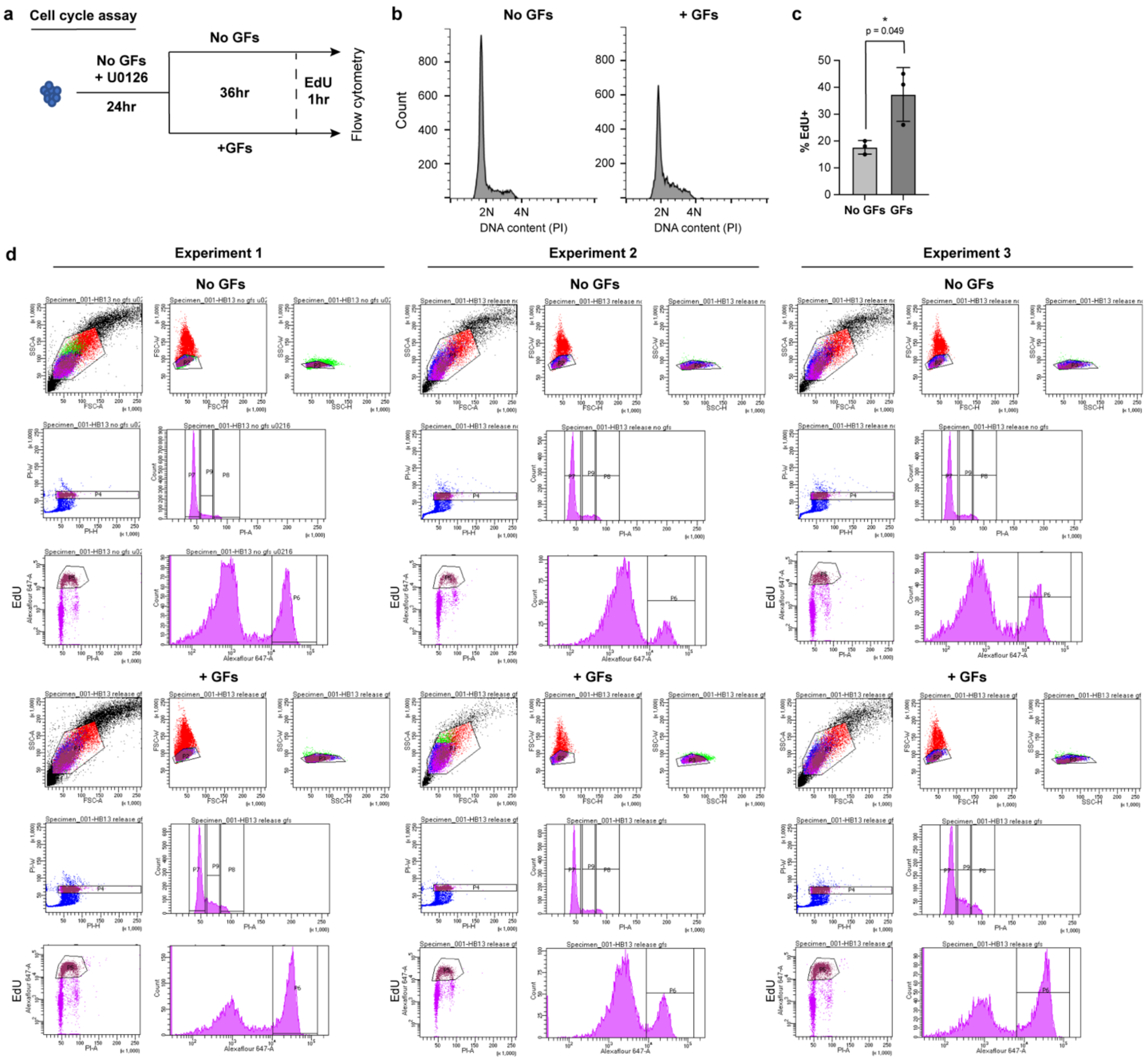

Supplementary Fig. S10 (related to Fig. 6). a) Schema for cell cycle assay in b) and c). b) Representative flow cytometry analysis of HB13 cells growing in media with no GFs and with U0126 (5 μM) for 24hrs, then released into media with no GFs or with all GFs for 36 hrs, stained with PI for DNA content. c) Quantification of %EdU+ cells from flow cytometry experiment on HB13 cells as in a) (mean and s.d., n = 3 independent experiments, * denotes p<0.05 by paired student t-test). d) Gating strategies for experiments in c). Source data are provided as a Source Data file.

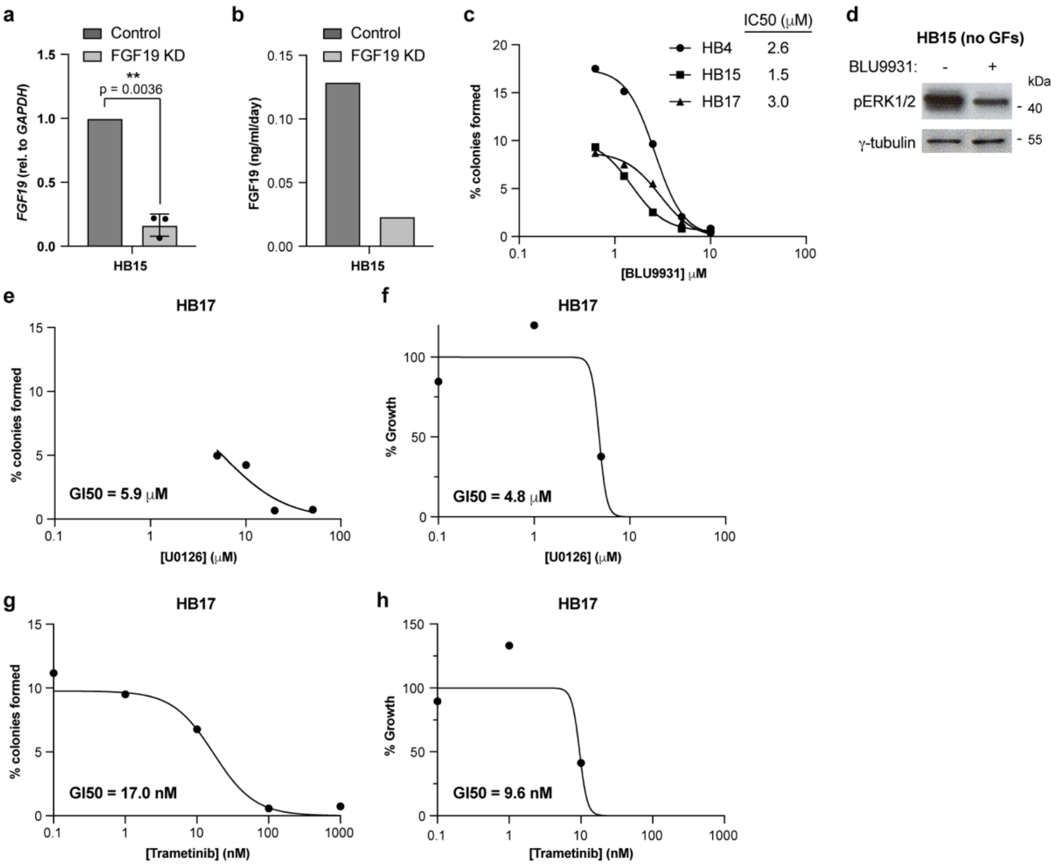

Supplementary Fig. S11 (related to Fig. 7). a) qRT-PCR for FGF19 in HB15 cells expressing lentiviral shRNA to luciferase (control) or *FGF19*. Mean and s.d., n = 3 independent experiments, paired two-tailed t-test, ** denotes p<0.01. b) FGF19 ELISA in HB15 cells expressing lentiviral shRNA to luciferase (control) or *FGF19*, grown in the absence of exogenous growth factors. c) Quantification of colony formation assay of single cells from the indicated tumoroids in media without GFs and with the FGFR4 inhibitor, BLU9931, at the concentrations indicated, with estimated IC50 as indicated. d) Immunoblot for phospho-ERK and control (γ-tubulin) in HB15 cells growing in media without exogenous growth factors, in the absence and presence of BLU9931 10 μM (2 hr). e) Quantification of colony formation assay of single cells from HB17 in media without GFs and with the MEK inhibitor, U0126, at the concentrations indicated, with estimated GI50. f) Quantification of % HB17 tumoroid growth after 3 days in media without GFs and with the MEK inhibitor, U0126, at the concentrations indicated, with estimated GI50. g) Quantification of colony formation assay of single cells from HB17 in media without GFs and with the MEK inhibitor, trametinib, at the concentrations indicated, with estimated

GI50. h) Quantification of % HB17 tumoroid growth after 3 days in media without GFs and with the MEK inhibitor, trametinib, at the concentrations indicated, with estimated GI50. Source data are provided as a Source Data file.

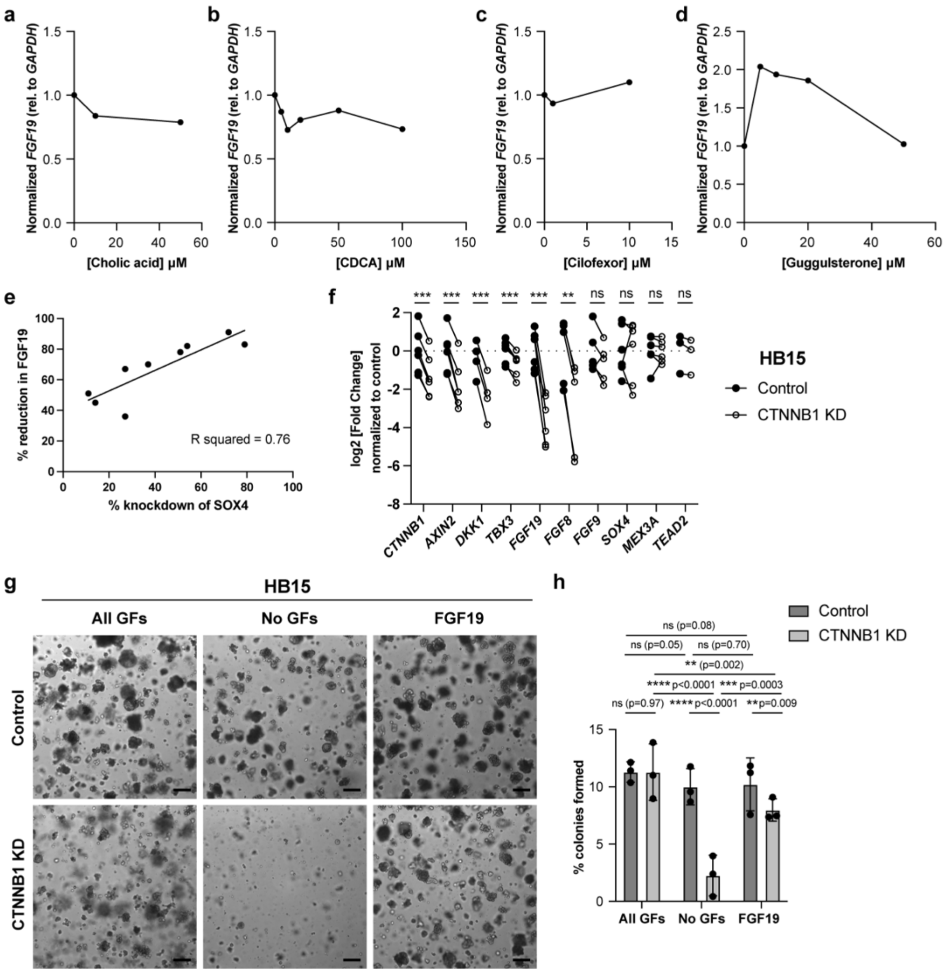

Supplementary Fig. S12 (related to Fig. 8). a) qRT-PCR for relative *FGF19* expression (normalized to *GAPDH*) in HB15 cells grown in media with all GFs after 24 hour incubation with cholic acid at the concentrations indicated. b) qRT-PCR for relative *FGF19* expression (normalized to *GAPDH*) in HB15 cells grown in media with all GFs after 24 hour incubation with chenodeoxycholic acid at the concentrations indicated. c) qRT-PCR for relative *FGF19* expression (normalized to *GAPDH*) in HB15 cells grown in media with all GFs after 48 hour incubation with FXR agonist, cilofexor, at the concentrations indicated. d) qRT-PCR for relative *FGF19* expression (normalized to *GAPDH*) in HB15 cells grown in media with all GFs after 48 hour incubation with FXR antagonist, guggulsterone, at the concentrations indicated. e) Graph showing % reduction in *FGF19* expression level as a function of % knockdown of *SOX4* as assessed by qRT-PCR across multiple experiments in which *SOX4* was knocked down by lentiviral shRNA in HB15 or HB17. f) qRT-PCR for Wnt target genes, FGFs, and SOX4 target genes in HB15 cells

expressing lentiviral shRNA to luciferase (control) or *CTNNB1*. Exact p-values, paired two-tailed t-test – *CTNNB1* (n=6 independent experiments): 0.000002, *AXIN2* (n=6): 0.0002, *DKK1* (n=4): 0.0009, *TBX3* (n=6): 0.0005, *FGF19* (n=6): 0.0003, *FGF8* (n=5): 0.0010, *FGF9* (n=5): 0.16, *SOX4* (n=6): 0.97, *MEX3A* (n=6): 0.95, *TEAD2* (n=3): 0.13. g) Brightfield images of HB15 colonies expressing lentiviral shRNA to luciferase (control) or *CTNNB1*, seeded as single cells after 3 days of antibiotic selection, and grown for 2 weeks in media containing EGF + FGF10 + HGF (All GFs), no GFs, or FGF19. Scale bar: 200 μm. h) Quantification of colony assay as in g). Mean and s.d., n=3 independent experiments, ANOVA with uncorrected Fisher's LSD. For all panels – *: p<0.05, **: p<0.01, ***: p<0.001, ns: not significant. Source data are provided as a Source Data file.
