# Supplementary material for: A developmental biliary lineage program cooperates with Wnt activation to promote cell proliferation in hepatoblastoma
Source: Nat Commun. 2024 Nov 20;15:10007. doi: 10.1038/s41467-024-53802-4 (PMC11579301; doi:10.1038/s41467-024-53802-4)
Supplement: Supplementary file 3 — Reporting Summary [file 41467_2024_53802_MOESM3_ESM.pdf]

nature portfolio

Corresponding author(s): Peng V. Wu, Roel Nusse

Last updated by author(s): Oct 13, 2024

Reporting Summary

Nature Portfolio wishes to improve the reproducibility of the work that we publish. This form provides structure for consistency and transparency in reporting. For further information on Nature Portfolio policies, see our Editorial Policies and the Editorial Policy Checklist.

Statistics

For all statistical analyses, confirm that the following items are present in the figure legend, table legend, main text, or Methods section.

n/a	Confirmed	
☐	☒	The exact sample size (*n*) for each experimental group/condition, given as a discrete number and unit of measurement
☐	☒	A statement on whether measurements were taken from distinct samples or whether the same sample was measured repeatedly
☐	☒	The statistical test(s) used AND whether they are one- or two-sided<br>*Only common tests should be described solely by name; describe more complex techniques in the Methods section.*
☒	☐	A description of all covariates tested
☐	☒	A description of any assumptions or corrections, such as tests of normality and adjustment for multiple comparisons
☐	☒	A full description of the statistical parameters including central tendency (e.g. means) or other basic estimates (e.g. regression coefficient) AND variation (e.g. standard deviation) or associated estimates of uncertainty (e.g. confidence intervals)
☐	☒	For null hypothesis testing, the test statistic (e.g. *F*, *t*, *r*) with confidence intervals, effect sizes, degrees of freedom and *P* value noted<br>*Give P values as exact values whenever suitable.*
☒	☐	For Bayesian analysis, information on the choice of priors and Markov chain Monte Carlo settings
☒	☐	For hierarchical and complex designs, identification of the appropriate level for tests and full reporting of outcomes
☐	☒	Estimates of effect sizes (e.g. Cohen's *d*, Pearson's *r*), indicating how they were calculated

Our web collection on statistics for biologists contains articles on many of the points above.

Software and code

Policy information about availability of computer code

Data collection	Tumoroid colony formation assay data were collected using the HCS Studio Cell Analysis Software. Flow cytometry data was collected with BD FACS Diva software. Gene expression data by qRT-PCR was collected using the StepOnePlus Real-Time PCR Software.
Data analysis	Differential expression analyses on RNA sequencing data from Smart-3SEQ were performed with DESeq2 version 1.38.3. The GSEA 4.3.2 application was used to run GSEAPreranked on gene lists obtained by DESeq2 ranked by padj. For 10x single cell RNA sequencing, sample demultiplexing, barcode processing, single-cell counting, and reference genome mapping were performed using the Cell Ranger Software version 3.0.2, with reference genome GRCh38.3.0.0. Dimensionality reduction by principal component analysis (PCA), graph-based clustering, and UMAP visualization were performed using Seurat version 4.3.0. Additional software used to analyze single cell RNA sequencing data includ scvelo, velocyto, Monocle. For spatial transcriptomics, Space Ranger software was used for downstream analysis and the Loupe Browser software was used for UMAP clustering and visualization. Fluorescence images were analyzed with TrackMate in ImageJ/Fiji.

For manuscripts utilizing custom algorithms or software that are central to the research but not yet described in published literature, software must be made available to editors and reviewers. We strongly encourage code deposition in a community repository (e.g. GitHub). See the Nature Portfolio guidelines for submitting code & software for further information.

Data

Policy information about availability of data

All manuscripts must include a data availability statement. This statement should provide the following information, where applicable:

- Accession codes, unique identifiers, or web links for publicly available datasets
- A description of any restrictions on data availability
- For clinical datasets or third party data, please ensure that the statement adheres to our policy

The metadata and transcript counts from Smart-3SEQ generated in this study are provided in Supplementary Data 1. Raw sequencing data from the Smart-3SEQ experiment in this study are available in the Gene Expression Omnibus (GEO) under accession code GSE279385 (https://www.ncbi.nlm.nih.gov/geo/query/acc.cgi?acc=GSE279385). Raw sequencing data from spatial transcriptomic and single cell RNA sequencing experiments in this study are available in GEO under accession code GSE249965 (https://www.ncbi.nlm.nih.gov/geo/query/acc.cgi?acc=GSE249965) and GSE233923 (https://www.ncbi.nlm.nih.gov/geo/query/acc.cgi?acc=GSE233923), respectively. Gene lists for specific signatures identified in this study and by prior single cell RNA sequencing and microarray studies on hepatoblastoma as well as single cell RNA sequencing of human fetal liver, all of which were used in this study, are provided in the Supplementary Data 5. Hepatoblastoma tumoroids are available upon request. Source data are provided as a Source Data file.

Research involving human participants, their data, or biological material

Policy information about studies with human participants or human data. See also policy information about sex, gender (identity/presentation), and sexual orientation and race, ethnicity and racism.

Reporting on sex and gender	Sex of patient specimens are reported in Supplementary Table S1 and S3. Sex was not taken into account in designing the study, however sex of patient specimens ultimately was representative of the general population of patients with hepatoblastoma.
Reporting on race, ethnicity, or other socially relevant groupings	Due to low patient numbers, in order to keep data anonymized, we do not report any information on race/ethnicity. Due to the low patient numbers, we could not make any conclusions about associations with race/ethnicity.
Population characteristics	Human patient information including age and sex are reported in Supplementary Table S1 and S3.
Recruitment	All patients treated with a diagnosis of hepatoblastoma at a single institution, Lucile Packard Children's Hospital, between 2003-2023 were identified. Research on these specimens was exempt from consent by the IRB due to their historical nature and impractical logistics. New patients diagnosed between 2018-2023 were approached to consent for fresh tissue collection. Patients were not otherwise prescreened, to avoid bias in selection.
Ethics oversight	The study protocol involving human subjects research was approved by the Stanford University Institutional Review Board.

Note that full information on the approval of the study protocol must also be provided in the manuscript.

Field-specific reporting

Please select the one below that is the best fit for your research. If you are not sure, read the appropriate sections before making your selection.

☒ Life sciences ☐ Behavioural & social sciences ☐ Ecological, evolutionary & environmental sciences

For a reference copy of the document with all sections, see nature.com/documents/nr-reporting-summary-flat.pdf

Life sciences study design

All studies must disclose on these points even when the disclosure is negative.

Sample size	No sample size calculation was performed prior to the study. The sample size was based on the availability of tumor specimens and tumoroids.
Data exclusions	No data were excluded.
Replication	All experiments were performed in specimens from at least two different patients. At least two independent biological replicates were performed for each experiment to replicate the data. All attempts at replication were successful. For statistical analyses, at least three independent biological replicates were performed, and these experiments included technical replicates if possible to verify assay consistency.
Randomization	When performing experiments, samples were split into different wells and randomly assigned to interventions vs. control.
Blinding	Investigators were not blinded to the identity of specimens/tumoroids/samples due to logistics as most experiments were performed by one individual and there were not enough personnel to make blinding logistically feasible. Analyses of sequencing data was performed in an unsupervised manner.

Reporting for specific materials, systems and methods

We require information from authors about some types of materials, experimental systems and methods used in many studies. Here, indicate whether each material, system or method listed is relevant to your study. If you are not sure if a list item applies to your research, read the appropriate section before selecting a response.

Materials & experimental systems

n/a	Involved in the study
☐	☒ Antibodies
☐	☒ Eukaryotic cell lines
☒	☐ Palaeontology and archaeology
☒	☐ Animals and other organisms
☒	☐ Clinical data
☒	☐ Dual use research of concern
☒	☐ Plants

Methods

n/a	Involved in the study
☒	☐ ChIP-seq
☐	☒ Flow cytometry
☒	☐ MRI-based neuroimaging

Antibodies

Antibodies used

The following antibodies were used for immunofluorescence: HNF4alpha (rabbit polyclonal, 1:50; Santa Cruz sc8987), KRT19 (rabbit, 1:100, Abbomax 602-670, clone nan), MKI67 (rat monoclonal, 1:100; eBioscience 14-5698-82, clone SolA15), phospho-histone H3 (ser10) (rabbit polyclonal, 1:1000, Millipore 06-570), beta-catenin (mouse FITC-conjugated, 1:50, BD beta-Catenin clone #14, custom AB #624044; detected with secondary antibody to FITC). The following antibodies were used for immunoblotting (diluted in 5% milk/PBST unless otherwise indicated): FGF19 (mouse monoclonal, 1 ug/ml, R&D MAB969), GFP (rabbit polyclonal, 1:5000, OriGene TA150122), phospho-ERK1/2 (rabbit polyclonal, 1:1000 in 1% BSA/TBST, Cell Signaling Technology 9101S), ERK1/2 (rabbit polyclonal, 1:1000, Cell Signaling Technology 9102S), gamma-tubulin (mouse monoclonal, 1:10,000, Abcam GTU-88 ab-11316), importin beta-1 (rabbit monoclonal, 1:2000, Cell Signaling Technology 60769S).

Validation

Validation for antibodies were provided in the manufacturers' websites:
HNF4alpha - https://www.scbt.com/p/hnf-4alpha-antibody-h-171
KRT19 - https://www.abbomax.com/product.aspx?id=609
MKI67 - https://www.thermofisher.com/antibody/product/Ki-67-Antibody-clone-SolA15-Monoclonal/14-5698-82
phospho-histone H3 - https://www.sigmaaldrich.com/US/en/product/mm/06570
FGF19 - https://www.rndsystems.com/products/human-fgf-19-antibody-117601_mab969
GFP - https://www.origene.com/catalog/antibodies/tag-antibodies/ta150122/rabbit-polyclonal-mgfp-antibody
phospho-ERK - https://www.cellsignal.com/products/primary-antibodies/phospho-p44-42-mapk-erk1-2-thr202-tyr204-antibody/9101
ERK1/2 - https://www.cellsignal.com/products/primary-antibodies/p44-42-mapk-erk1-2-antibody/9102
gamma-tubulin - https://www.abcam.com/en-us/products/primary-antibodies/gamma-tubulin-antibody-gtu-88-centrosome-marker-ab11316
importin beta-1 - https://www.cellsignal.com/products/primary-antibodies/importin-b1-e1f1g-rabbit-mab/60769
Validation of the beta-catenin antibody was performed by detecting absence of the protein via immunofluorescence following shRNA knockdown in hepatoblastoma cells. FGF19 antibody was validated in this study after overexpressing FGF19 in HB12 cells and detecting FGF19 as in Fig. 6g.

Eukaryotic cell lines

Policy information about cell lines and Sex and Gender in Research

Cell line source(s)

293T cells were obtained from ATCC (CRL-3216). The information for all human hepatoblastoma tumoroids in this manuscript are provided in Supplementary Table S3. Parents of all patients treated at Lucile Packard Children's Hospital with a presumed diagnosis of liver tumor were approached before biopsy or surgery to discuss informed consent for tissue banking, including collection of live cells for tumoroid generation. There were no selection biases since all eligible patients' parents were approached if available.

Authentication

Cell lines were not authenticated.

Mycoplasma contamination

Cell lines were not tested for Mycoplasma contamination.

Commonly misidentified lines
(See ICLAC register)

None.

Plants

Seed stocks	*Report on the source of all seed stocks or other plant material used. If applicable, state the seed stock centre and catalogue number. If plant specimens were collected from the field, describe the collection location, date and sampling procedures.*
Novel plant genotypes	*Describe the methods by which all novel plant genotypes were produced. This includes those generated by transgenic approaches, gene editing, chemical/radiation-based mutagenesis and hybridization. For transgenic lines, describe the transformation method, the number of independent lines analyzed and the generation upon which experiments were performed. For gene-edited lines, describe the editor used, the endogenous sequence targeted for editing, the targeting guide RNA sequence (if applicable) and how the editor was applied.*
Authentication	*Describe any authentication procedures for each seed stock used or novel genotype generated. Describe any experiments used to assess the effect of a mutation and, where applicable, how potential secondary effects (e.g. second site T-DNA insertions, mosiacism, off-target gene editing) were examined.*

Flow Cytometry

Plots

Confirm that:

☒ The axis labels state the marker and fluorochrome used (e.g. CD4-FITC).

☒ The axis scales are clearly visible. Include numbers along axes only for bottom left plot of group (a 'group' is an analysis of identical markers).

☒ All plots are contour plots with outliers or pseudocolor plots.

☒ A numerical value for number of cells or percentage (with statistics) is provided.

Methodology

Sample preparation	Tumoroids were incubated in media with no growth factors and U0126 5 M for 24 hours. Media was removed and tumoroids were washed twice with media without growth factors or drug and replaced with either media containing growth factors or media without growth factors. After 35 hours, EdU 10 microM was added for 1 hour, then tumoroids were isolated from Matrigel using dispase and further dissociated to single cells using TrypLE. Single cells were washed with liver perfusion media with 5% FBS and fixed with 70% ethanol. Cells were stained with the Click-iT Plus EdU Alexa Fluor 647 Flow Cytometry Assay Kit (Thermo Fisher C10340) and propidium iodide for DNA content.
Instrument	Flow cytometry was performed on a BD FACS Aria II machine.
Software	FlowJo was used for analysis.
Cell population abundance	Not applicable as cells were only analyzed and not sorted.
Gating strategy	Standard methods to identify FSC/SSC gates were used to exclude doublets and dead cells. Gating strategy is included in Supplementary Fig. S10.

☒ Tick this box to confirm that a figure exemplifying the gating strategy is provided in the Supplementary Information.
