# Supplementary material for: A developmental biliary lineage program cooperates with Wnt activation to promote cell proliferation in hepatoblastoma
Source: Nat Commun. 2024 Nov 20;15:10007. doi: 10.1038/s41467-024-53802-4 (PMC11579301; doi:10.1038/s41467-024-53802-4)
Supplement: Supplementary file 4 — Description of Additional Supplementary Files [file 41467_2024_53802_MOESM4_ESM.pdf]

SUPPLEMENTARY DATA

A developmental biliary lineage program cooperates with Wnt activation to promote cell proliferation in hepatoblastoma

Peng V. Wu[1,2,3,4,5,6]*, Matt Fish[1,2,3], Florette K. Hazard[7,8], Chunfang Zhu[7], Sujay Vennam[7], Hannah Walton[1,2,3,9], Dhananjay Wagh[10], John Coller[10], Joanna Przybyl[7,11,12], Maurizio Morri[13,14], Norma Neff[14], Robert B. West[7], and Roel Nusse[1,2,3]*

[1] Howard Hughes Medical Institute, Stanford University School of Medicine, Stanford, CA 94305, USA
[2] Department of Developmental Biology, Stanford University School of Medicine, Stanford, CA 94305, USA
[3] Institute for Stem Cell Biology and Regenerative Medicine, Stanford University School of Medicine, Stanford, CA 94305, USA
[4] Department of Pediatrics, Stanford University School of Medicine, Stanford, CA 94305, USA
[5] Current affiliation: Division of Oncology, Cincinnati Children's Hospital Medical Center, Cincinnati, OH 45229, USA
[6] Current affiliation: Department of Pediatrics, University of Cincinnati College of Medicine, Cincinnati, OH 45229, USA
[7] Department of Pathology, Stanford University School of Medicine, Stanford, CA 94305, USA
[8] Current affiliation: Department of Pathology and Laboratory Medicine, University of California Davis School of Medicine, Sacramento, CA 95817 USA
[9] Current affiliation: Department of Population Health, NYC Health + Hospitals, New York, NY 10004 USA
[10] Stanford Genomics, Stanford University, Stanford, CA 94305, USA
[11] Current affiliation: Department of Surgery, McGill University, Montreal, H4A 3J1 QC, Canada
[12] Current affiliation: Cancer Research Program, The Research Institute of the McGill University Health Centre, Montreal, H4A 3J1 QC, Canada
[13] Chan Zuckerberg Biohub, Stanford, CA 94305, USA
[14] Current affiliation: Altos Labs, Redwood City, CA 94065, USA

*Correspondence to: Peng.Wu@cchmc.org and rnusse@stanford.edu

Source Data File. Source data for primary figures and supplementary figures

Supplementary Data 1. Metadata and Smart-3SEQ results for 72 micro-dissected hepatoblastoma specimens

Supplementary Data 2. Genes differentially expressed in embryonal, fetal, and mesenchymal components of hepatoblastoma, compared to normal liver and to each other, as determined by DESeq2

Supplementary Data 3. Cluster marker genes identified by spatial transcriptomics of primary hepatoblastomas

Supplementary Data 4. Single cell RNA sequencing of hepatoblastoma tumoroids: QC, cell number, and marker genes for each cluster

Supplementary Data 5. Gene lists used to overlay gene signature scores on tumoroid scRNAseq data
